# New Zealand’s Approaches to Regulating the Commodification of the Female Body: A Comparative Analysis Reveals Ethical Inconsistencies

**DOI:** 10.1007/s11673-023-10246-7

**Published:** 2023-04-05

**Authors:** Lauren S. Otterman

**Affiliations:** University of Otago Bioethics Centre, 71 Frederick Street, Dunedin, 9016 New Zealand

**Keywords:** Prostitution, Surrogacy, Assisted reproductive technologies, Commodification, Bodily ownership, Sex workers’ rights, HART Act, PRA

## Abstract

In 2003 and 2004, Aotearoa New Zealand enacted two key laws that regulate two very different ways in which the female body may be commodified. The Prostitution Reform Act 2003 (PRA) decriminalized prostitution, removing legal barriers to the buying and selling of commercial sexual services. The Human Assisted Reproductive Technology Act 2004 (HART Act), on the other hand, put a prohibition on commercial surrogacy agreements. This paper undertakes a comparative analysis of the ethical arguments underlying New Zealand’s legislative solutions to prostitution and commercial surrogacy. While the regulation of prostitution is approached with a Marxist feminist lens with the aim to ensure the health and safety of sex workers, commercial surrogacy is prohibited outright for concerns of negative impacts on present and future persons. I ground the principles of each Act in their ethical foundations and compare these two against one another. I conclude that New Zealand’s legislative approach to regulating the commodification of the female body is ethically inconsistent.

## Introduction

In 2003 and 2004, Aotearoa New Zealand enacted two laws that regulate two of the most controversial and intimate aspects of human life. The Prostitution Reform Act 2003 (hereafter “the PRA”) decriminalized prostitution, removing legal barriers to the buying and selling of commercial sexual services. The Human Assisted Reproductive Technology Act 2004 (hereafter the “HART Act”), on the other hand, put a prohibition on commercial surrogacy agreements. Both seek to regulate the commodification of aspects of the female body but go about it in diverse ways that have quite distinct outcomes.

The PRA created a legal framework for decriminalized prostitution with a two-fold purpose to safeguard the rights of sex workers and promote their welfare and occupational health and safety (s 3(a)-(b)). This came after years of lobbying by sex workers’ rights activists, spearheaded by the New Zealand Prostitutes Collective (NZPC) (Barnett et al. 2010). Other jurisdictions with similar goals of promoting public health and gender equality, notably Sweden and other Nordic countries, have used a very different approach. Aptly known as the “Nordic Model” or the “Equality Model,” this model views prostitution as an inherently harmful and inescapably gendered practice; as long as men have access to women’s bodies as a commodified good, gender equality is unachievable. The Nordic Model thus decriminalizes the selling of sex while criminalizing the purchase of sex, aiming to reduce the demand for commercial sex that fuels the industry.

The passing of the PRA in New Zealand made the commodification of sexual services legally permissible, subject to certain safety and labour regulations. This was heralded a great victory by sex workers’ rights groups like NZPC who saw decriminalization as the first step away from the harmful social stigmatization of sex workers and towards a safer and more equitable working environment (Barnett et al. 2010). At roughly the same time, Parliament was tasked with crafting a legislative regime to regulate a wave of new reproductive technologies that had emerged in the twenty-five years since the birth of Louise Brown, the world’s first “IVF baby,” in 1978. Through IVF and gestational surrogacy, it became possible for individuals or couples unable to become pregnant to have biologically related children by utilizing a surrogate for gestational services. A question facing legislators was how to conceptualize, and put boundaries around, the relationship between gestational surrogate and commissioning parents.

The HART Act’s primary purpose, stated in section 3(a), is “to secure the benefits of assisted reproductive procedures … by taking appropriate measures for the protection and promotion of the health, safety, dignity, and rights of all individuals, but particularly those of women and children.”^1^ The most relevant section for our purposes is section 14, which provides that it is an offense to “give or receive valuable consideration” to any party in a surrogacy arrangement. While the HART Act explicitly shares a core principle with the PRA—to promote the health and safety of individuals involved in practice in question, assisted reproduction and prostitution, respectively—legislators declared it unlawful to pay a surrogate for their services, while just a year before they had removed legal barriers to purchasing sexual services from sex workers. It is interesting that the argument that the health and safety of sex workers should be protected in whatever work they choose, so successful in passing the PRA, did not gain any traction when applied to reproductive labour.

In this essay, I will interrogate whether these opposing regulatory approaches are ethically consistent. I begin by analysing the purposes and principles of the two Acts in order to identify the ethical arguments that were successful in influencing New Zealand’s regulation of prostitution on one hand, and surrogacy on the other. I argue that these two views are fundamentally in conflict by illustrating how different the regulation of prostitution and surrogacy would look if their current ethical foundations were reversed. I end by analysing the two respective ethical standpoints to determine whether the legislation governing the acceptable versus unacceptable commodification of the female body is consistent. I conclude that the ethical foundations for each approach are inconsistent.

Before proceeding, it should be noted that there are many key differences between sexual and reproductive labour. For one, gestational surrogates do not have any sexual interaction with the commissioning parent(s), while sex workers are paid to perform sexual services for their clients, although this does not always include sexual intercourse. A second key difference is that while clients engage sex workers for sexual pleasure or companionship, the desired outcome of surrogacy agreements is the birth of a child. However, I will argue that both sex work and gestational surrogacy can reasonably be viewed as types of bodily labour that should be regulated in a similar manner.

## The Ethical Argument for New Zealand’s Regulation of Prostitution

There is a history of lively debate on how to best approach the problem of prostitution, especially among feminist scholars (e.g., Overall 1992; Sullivan 1995; Sanders 2016; Senent Julián 2019; Shamir 2019). In New Zealand, decriminalization was a controversial approach, passing in Parliament by only one vote. It was, however, the model proposed by the most vocal sex workers’ rights group, NZPC, who were very influential in lobbying for this legislative change (Barnett et al. 2010). The full decriminalization approach to prostitution takes the feminist-Marxist view that sex work is simply one of many forms of labour exploited under a capitalist system (Tremblay 2021). This view holds that sex workers are made vulnerable not because of the nature of their work qua sex work but the fact that a criminalized environment leaves them vulnerable to exploitation and violence from sex buyers. They are also denied access to standard public health and workplace safety standards, struggle to access equal treatment under the law, and may face housing and hiring discrimination due to their occupation (Jordan 2020).

Solutions proposed by sex workers’ rights groups focus on decriminalizing sex workers in order to allow them to continue working but in safer conditions. They argue that such changes reduce social stigma against sex workers and empower them to take more control over which services they provide, and to whom, without fear of entrapment by police (Jordan 2020). Under more restrictive regimes, sex workers report being “wary of reporting labour rights violations or assaults because of the fear of being treated poorly as sex workers” (Easterbrook-Smith 2020, 162). A full decriminalization policy is grounded in the understanding of prostitution primarily as an economic activity that “involves choice for many participants but vulnerability and exploitation for some” (Benoit et al. 2019, 1916). For example, NZPC note that under the prior legislative regime of full criminalization Māori street-based sex workers were the greatest targets of police action, comprising 70 per cent of convictions for soliciting despite comprising only 15 per cent of New Zealand’s general population (Healy, Pickering, and Hati 2020, 42). Therefore, from the perspective of NZPC, echoed by a diverse range of feminist groups in submissions to Parliament, decriminalizing prostitution would improve working conditions for all sex workers but especially those already marginalized such as transgender and Māori sex workers (Healy, Pickering, and Hati 2020, 49; New Zealand Prostitutes Collective 2013).

The purpose of the PRA, as stated in section 3 is “to decriminalise prostitution … and to create a framework that—(a) safeguards the human rights of sex workers and protects them from exploitation.” The Act does not expand on which specific human rights it is seeking to safeguard on behalf of sex workers. However, based on successful arguments made by NZPC, a relevant human right in this context might be “the right to work, to free choice of employment, to just and favourable conditions of work and to protection against unemployment,” under Article 23 of the United Nations *Universal Declaration of Human Rights* (United Nations General Assembly 1948)*.* This argument applied to sex work can be expressed by the syllogism:

- P1: Everyone has the right to free choice of employment and just and favourable conditions of work.
- P2: Sex work is work.
- C: Individuals have the right to freely choose to work in the commercial sex industry in just and favourable conditions.

Embedded in the sex workers’ rights view is the key assumption that individuals engaging in sex work are freely choosing to do so. This is a central point of contention between those lobbying for decriminalization on one hand and those on the neo-abolitionist side who question just how freely many individuals in the commercial sex industry actually choose to be there. This view will be explored in more depth below. 

Groups lobbying for decriminalization make surprisingly little mention of the violence and exploitation of the sex in sex work. This omission, Showden suggests, strengthens the critique of capitalist structures and makes it “easier to highlight what’s exploitative about the *work* as opposed to emphasizing the moral quandary of sex work” (2020, 68). This suggests a strategic move to situate the decriminalization discussion firmly in the realm of labour rights rather than opening even more ethically fraught conversations around bodily ownership and commodification, sexual liberty, and structures of gendered violence, among others.

The sex workers’ rights position can be summed up simply: “sex workers need to be safe, their labour protected by labour laws, and this will not happen until we truly listen to those that matter the most—the labourers” (Showden 2020, 103). This last note suggests that the question of how best to deal with prostitution could be resolved if we just asked those in the industry. Research on people in prostitution, like research on other groups engaging in stigmatized or illegal practices, suffers from methodological issues (Jordan 2005). Proponents of the sex workers’ rights view and the opposing neo-abolitionist stance have each reported empirical interview data that aligns with their perspectives, leading to literature rife with contradiction. Coy, Smiley, and Tyler point out an uncomfortable truth that balanced and representative samples of sex workers’ experiences in prostitution research are exceptionally difficult to capture because “there are many women who will not share their experiences or analyses because they choose not to and, in some cases, because they are no longer alive" (2019, 1933).

The difficulty of interpreting literature is further complicated when the same document is even read in diametrically opposing ways. This is demonstrated by looking at differing interpretations of one key document: the Prostitution Law Review Committee’s statutorily mandated five-year review of the impacts of the PRA, *Report on the Operation of the Prostitution Reform Act 2003* (hereafter “*the Report*”). Both Moran and Farley (2019) and Waltman (2011) cite this report as evidence of the inefficacy of decriminalization, that the majority of sex workers felt that the law could do little about violence that occurred. On the other hand, Healy, Pickering, and Hati also mention the same report stating that it “produced good evidence-based research that supported the success of the Act” (2020, 48). I suggest that one possible reason for this difference in interpretation stems from the different end goals of each side. Voices from NZPC stress the *relative* improvements in safety and working conditions after the PRA compared with before. *The Report* contains ample evidence to support this claim, including the fact that on average 65 per cent of sex workers interviewed felt more able to refuse a client since the law change and “numerous examples of sex workers being able to negotiate safer sex by stating that it is against the law for them not to practice it” (Prostitution Law Review Committee 2008, 50). Neo-abolitionists such as Moran and Farley, on the other hand, likely view *the Report* through an *absolute* lens. For example, while it might be reported as a success that *only* 3 per cent of sex workers reported being raped by a client (Prostitution Law Review Committee 2008, 56), and *only* 3.9 per cent of people were made to work by a third party (Prostitution Law Review Committee 2008, 63), these still represent the experiences of hundreds of people, based on estimates that there between 3,500 (Abel, Fitzgerald, and Brunton 2009) and 5,932 (Prostitution Law Review Committee 2005) sex workers in New Zealand.^2^ The existence of *any* abuse or violence whatsoever proves that the commercial sex industry is harmful and exploitative and should not be tolerated.

Showden (2020) argues that decriminalization is the first step in a sex-worker-led feminism that can focus on the structural causes of poverty that limit educational and economic opportunities and thus leave sex work as the only option for many people. She asserts that we can use our “current heightened attention to the pervasiveness of gendered sexual violence” as a way to shift the focus away from sex work per se and more onto the aspects that make it a harmful industry: masculinity, power, and money (Showden 2020, 71). This work, then, would also have correlate impacts on the other forms of sexual violence faced by women and gender diverse people in many other types of workplaces and in their homes.

A barrier to having these important conversations, Armstrong and Fraser (2020) assert, is the social stigma of sex work. The pervasive belief that sex workers are either criminals (under a fully criminalized model) or victims of exploitation and abuse by violent clients (under a partial decriminalization model such as the Nordic model) is a barrier to productive conversations about how best to improve the rights and safety of sex workers. Furthermore, social stigma puts sex workers in a double bind where sex work is perceived as more acceptable when it is financially lucrative and the work enjoyable. They thus risk whatever tenuous social acceptability they have if they voice dissatisfaction with their working conditions as either financially suboptimal or unsafe (Easterbrook-Smith 2020). Removing social stigma through decriminalization is then an important step towards improving the visibility of sex workers as a population and addressing their access to justice and health and safety needs.

### This Approach Applied to Commercial Surrogacy

As explored above, the argument core to the decriminalization of sex work under the PRA focused on sex work as nothing more than work. While there are some risks involved that are quite specific to the commercial sex industry, under the sex workers’ rights view, if someone chooses to use their body for work, they should be free to do so, in safe conditions. We can apply this reasoning to the case of commercial surrogacy. The argument might look as follows:

- P1: Everyone has the right to free choice of employment and just and favourable conditions of work.
- P2: Gestational surrogacy is work.
- C: Individuals have the right to freely choose to work as gestational surrogates in just and favourable conditions.

This view is analogous to the sex worker’s rights view of prostitution because it holds that surrogacy is simply one of many forms of labour, and still only one of many forms of truly *embodied* labour. It is not morally significantly different from other forms of work such as caring for children or elderly parents, nursing, or, for some, prostitution. Furthermore, this view highlights that in our capitalist economy when the “advancing frontier of commodification elicits recoil” (Showden 2020, 72) we should recognize that a desire to protect things that some perceive as inalienable from commodification does not change that they are for sale. To deny this reality is to live in an overly moralistic and imaginary world. Bodily ownership under this view is equivalent to property ownership and thus women should be empowered to do with their bodies what they choose, including selling aspects or abilities of it for a fee.

Surrogacy becomes problematic when it is compounded by various forms of power disparities, which some see as inevitable. Dorothy Roberts (2009, 799) posits that “the neoliberal reification of market logic is likely to expand the hiring or poor and low-income women of colour for their reproductive services.” This very situation occurs in many transnational surrogacy arrangements when middle-class, Western couples contract poorer women of colour in developing countries for their gestational services (Deonandan 2015).

This stark differential in wealth and status between the intended parents and gestational surrogate is one of many elements that can make commercial surrogacy arrangements exploitative. One argument may be that the exchange of money in itself is coercive and exploitative. However, in commercial surrogacy agreements, the exchange of money plays another critical role: determining who the baby’s “real” parents are. Since time immemorial, while fatherhood could always be questioned, there could be no doubt that a baby’s mother was the woman who gestated and gave birth to it. Lewis (2016, 196) notes how quickly this historically unquestioned reality has been subverted:

… the very fact that it is the gestator, rather than the person who steps in to be the parent, that is indexed as “surrogate,” is wholly contingent on the narratives of biogenetic property in the family, narratives that can be changed.

The ancient, deeply internalized norm of parenthood has been uprooted in the last fifty years with the dawn of IVF and gestational surrogacy. The institution of surrogacy relies on the central agreement that the “gestator” will agree to relinquish all parenting rights to the intended parents.

The main concern under this view, then, is that women can engage in surrogacy agreements free from exploitation, not unlike the concerns for safe working conditions for sex workers raised by NZPC under their sex workers’ rights view. While the HART Act does not prohibit surrogacy arrangement in themselves (s 14(1)), it does prohibit the giving or receiving of “valuable consideration” to participation in a surrogacy arrangement (s 13(3)). Those individuals who want to work as gestational surrogates, then, must do so altruistically without the expectation of being compensated for their work. This conclusion would appear grossly unjust if applied in the context of sex work: a sex worker may choose to provide sexual services but should not expect to be compensated for their work. The only way to avoid this unjust conclusion highlighted in the context of surrogacy would be to refute one of the two premises set out above. In the following section, I will explore the ethical argument underlying the prohibition of commercial surrogacy in New Zealand. In the subsequent section, I will then argue that it is the second premise, “gestational surrogacy is work” that is rejected. If surrogacy is not work, those engaging in surrogacy arrangements are not subject to the same protections under the human rights framework utilized to justify the decriminalization of prostitution.

## The Ethical Argument for New Zealand’s Regulation of Commercial Surrogacy

The first principle of the HART Act, under section 4(a), is that “the health and well-being of children born as a result of the performance of an assisted reproductive procedure or an established procedure should be an important consideration in all decisions about that procedure.” The second principle, stated in section 4(b) is: “the human, health, safety, and dignity of present and future generations should be preserved and promoted.” We can reasonably assume, then, that the prohibition of commercial surrogacy and other “commercial transactions relating to human reproduction” (s 3(c)) is motivated by concern for the health and well-being of the resultant children, perhaps even more so than for the individuals involved in the surrogacy agreement. Let us set this up as a syllogism:

- P1: Commercializing transactions relating to human reproduction is harmful to the health, safety, and dignity of present and future generations.
- P2: Harmful practices should be prohibited.
- P3: Surrogacy is a transaction relating to human reproduction.
- C: Commercial surrogacy should be prohibited.

Implicit in the HART Act, and explicit here, is that surrogacy arrangements are not work but a transaction related to human reproduction. In fact, treating surrogacy as work by giving or receiving valuable consideration is directly prohibited by the Act. As such, surrogacy is treated as a separate class of transactions subject to the specific regulations under the HART Act. Why is it that a “transaction relating to human reproduction” deserves greater protections than the purchase of sex?

Some authors have argued that there is no morally significant difference between prostitution and surrogacy. For example, Patrone (2018) sees surrogacy arrangements as a form of “reproductive prostitution.” Her Kantian view holds that it is morally impermissible to use a person as mere means to an end, be it sexual gratification or new life. Both prostitution and surrogacy breach the Kantian view of the absolute unity of the human person which asserts that we cannot conceptually separate the use of a person’s sexual or reproductive organs from use of that whole human being. The Kantian approach holds that an intuition that commercial surrogacy is more morally acceptable than prostitution may stem from our widely held belief that it is a joy for a child to be born to a loving family; however, even this cannot justify the treating of a woman’s body as mere means to that end.

Viewing the body as a whole entity echoes a central tenet of feminist bioethics that bodily ownership should be viewed as a relationship of mutual belonging and constitution rather than ownership (Mackenzie 2010). Under this view, the body is more than just one separable piece of the self to be utilized for an end. The body *is* the self. The constitutive approach to bodily ownership is consistent with Radin’s (1987) concept of market inalienability: the inseparability of the self from certain bodily “goods” that might be desirable market commodities, such as access to a woman’s body for sex or procreation.

It is interesting that this Kantian approach doesn’t appear to distinguish commercial surrogacy from altruistic surrogacy. The harm comes not from the commodification of gestation per se but on the process that precedes it: the disunification of a woman that turns her into a collection of parts. This leads me to conclude that, under the Kantian view, surrogacy becomes no more permissible even when a woman freely consents to an altruistic surrogacy arrangement. At its best, this act of generosity and altruism still constitutes “a strange splitting that looks much more life self-objectification” (Belu 2017, 48) whereby the act of conceptual splitting denies the reality that the woman is a unified whole in herself and, for the duration of her pregnancy, irremovably intertwined with the foetus in her uterus. As such, under this view, all forms of surrogacy are harmful to women and should not be permitted.

In New Zealand, however, altruistic surrogacy is legally permissible. Therefore, the primary harm to be avoided must not be that of a metaphysical splitting of the surrogate. What about the commercial element is specifically harmful? In fact, during public submissions on the draft legislation, only two submissions were received regarding surrogacy, both arguing *against* the prohibition of commercial surrogacy. The submissions argued that a woman should have the right to choose whether or not to financially benefit from utilizing her reproductive abilities for others (Park, McLaughlan, and Frengley 2008, 25). While the direct comparison to sex work was not mentioned in the submission, the reasoning used to argue for a decriminalized model of sex work echoes loudly here.

There was no documentation brought to regulators of widespread public animosity towards the commercialization of reproduction. Why, then, did regulators opt for a blanket prohibition on the commercialization of surrogacy and gamete or embryo donation? The harm regulators sought to prevent, then, must be harm to the children who would result from a commercial surrogacy arrangement. Without evidence of long-term physical or psycho-social impacts on such children (Barnes et al. 2004), was this restriction on individuals’ ability to reproduce and work as they choose justified? If we focus purely on the impacts on those acting as surrogates, this first view aligns with the neo-abolitionist view of prostitution, explored below, in that it sees the practice of commercial surrogacy as inherently harmful.

### This Approach Applied to Prostitution

We can apply this reasoning to the practice of prostitution:

- P1: Commercializing transactions relating to human sexual relationships is harmful to the health, safety, and dignity of present and future generations.
- P2: Harmful practices should be prohibited.
- P3: The purchase of sexual services is a transaction relating to human sexual relationships.
- C: The purchase of commercial sexual services should be prohibited.

These principles align with the views of “neo-abolitionist” feminists who see prostitution as “predominantly caused by hierarchal gender relations” (Benoit et al. 2019, 1907). This view holds that all forms of prostitution are inherently exploitative and represent “the absolute embodiment of patriarchal male privilege” (Kesler 2002), and as such prostitution should not be tolerated as an institution. The sex industry is driven by men’s demand for commodified sex which is normalized and perpetuated by the existence of the industry. Authors argue that this “normalization of purchasing sexual access to women has especially harmful consequences for particular groups of marginalized women, as well as having broader effects on the status of women as a class” (Coy, Smiley, and Tyler 2019, 1933). This view acknowledges that while men and trans people also engage in sex work, the majority of sex workers identify as women while buyers of sex are overwhelmingly men. As Waltman astutely points out, “the gender disparity in using and being used in prostitution is not complex and should be theoretically and empirically addressed—not evaded” (2011, 455).

Neo-abolitionists also stress how histories of victimization by family members and intimate partners make some more vulnerable to recruitment into the commercial sex industry. Proponents of this view also tend to focus on the high rates of physical and sexual violence faced by sex workers during their time in prostitution. For example, one multinational study of 854 people in prostitution found that 71 per cent were physically assaulted in prostitution; 63 per cent were raped; and 89 per cent would leave prostitution if they could but had no other options to survive (Farley et al. 2004, 34).

For Moran and Farley, the sex in sex work is always unwanted and coerced, and yet many champions of a woman’s right to sell her sexual services obscure this fact:

… unwanted sex in every other conceivable scenario is identified as sexually abusive. It is only in prostitution that the abusive nature of the sex is denied, and it is denied because the coercion itself is not identified. Prostitution will never be recognized as sexual abuse *until the cash transaction integral to it is identified as coercive* by its nature. (2019, 1950, my italics)

The assertion that monetary exchange constitutes coercion leads neo-abolitionists to reject the notion that sex work is really a free choice for many in the commercial sex industry.

Age is another relevant compounding factor that makes many vulnerable to victimization; as such it is seen as universally abhorrent to solicit commercial sex from a child. This view is ratified by Article 3 of the *Protocol to Prevent, Suppress and Punish Trafficking in Person, Especially Women and Children:*

The recruitment, transportation, transfer, harbouring or receipt of a child for the purpose of exploitation shall be considered “trafficking in persons” even if this does not involve any of the means set forth in subparagraph (a) of this article.^3^ (United Nations General Assembly 2000)

Therefore, any minors involved in prostitution are *by definition* victims of trafficking, regardless of how they find themselves in the industry.

Neo-abolitionists hold that prostitution and trafficking are inseparable issues, stemming from and perpetuated by the same patriarchal entitlement to control and access women’s bodies. This is not to say that all women in prostitution are victims of trafficking, but it does highlight the moral inconsistency in views that denounce trafficking but enthusiastically argue for less restrictive prostitution policies like decriminalization, particularly when it has been shown that many sex workers enter the sex industry as minors. The United States Office to Monitor and Combat Trafficking in Persons’ *2021 Trafficking in Persons Report: New Zealand* found that “while the government [of New Zealand] convicted offenders in more cases of child sex trafficking than in previous years, it did not identify any victims in these cases as trafficking victims” (U.S. Department of State 2021, ¶1). There appears to be a lack of understanding of, or reluctance to look at, the intimate link between the illegal trafficking of children and prostitution in a decriminalized setting in New Zealand despite evidence that a portion of sex workers entered the industry as minors.

One study of the demographics of sex workers in New Zealand found that an estimated 9.3 per cent of sex workers entered the industry aged between sixteen and seventeen, while another 9 per cent were under the age of sixteen when they entered the industry (Abel and Fitzgerald 2008, 264). Until their eighteenth birthday, these adolescents were trafficking victims. As Jackie Turner shrewdly points out, “prostitution, it seems, can be tolerated, but the means of delivering women into prostitution cannot” (2012, 53).

Neo-abolitionist feminists propose partial decriminalization policies such as the Nordic Model that focus on reducing the demand for commercial sex by criminalizing buyers of sex and third parties. It decriminalizes the sale of sex, instead offering sex workers and trafficking victims alike social support services like counselling, affordable housing, and job training, directed at helping them exit the sex industry. This model acknowledges many individuals in the commercial sex industry do not choose to be there, if we define choice as picking among more than one feasible option.

## Arguments for Regulating Prostitution and Commercial Surrogacy in New Zealand Are Ethically Inconsistent

Let us review the conclusions at which we have arrived through these analyses. I first set up the following syllogism to represent the ethical argument for the decriminalization of prostitution under the PRA:

- P1: Everyone has the right to free choice of employment and just and favourable conditions of work.
- P2: Sex work is work.
- C: Individuals have the right to freely choose to work in the commercial sex industry in just and favourable conditions.

I then explored how the regulation of surrogacy might look if it followed consistently from this ethical reasoning, using the argument:

- P1: Everyone has the right to free choice of employment and just and favourable conditions of work.
- P2: Gestational surrogacy is work.
- C: Individuals have the right to freely choose to work as gestational surrogates in just and favourable conditions.

These two arguments focus on the human rights of all individuals to free choice of employment and to just and favourable working conditions. Crucially here, both sex work and gestational surrogacy are conceptualized as simply forms of work and as such, those who choose to engage in either should be protected in their rights to make that choice, to be safe to do so, and justly compensated.

These two arguments, however, stand in stark contrast with the ethical reasoning extracted from the principles of the HART Act, where commercial surrogacy is prohibited based on such reasoning:

- P1: Commercializing transactions relating to human reproduction is harmful to the health, safety, and dignity of present and future generations.
- P2: Harmful practices should be prohibited.
- P3: Surrogacy is a transaction relating to human reproduction.
- C: Commercial surrogacy should be prohibited.

As another intimate form of embodied labour, prostitution could reasonably be conceptualized similarly, using this parallel argument:

- P1: Commercializing transactions relating to human sexual relationships is harmful to the health, safety, and dignity of present and future generations.
- P2: Harmful practices should be prohibited.
- P3: The purchase of sexual services is a transaction relating to human sexual relationships.
- C: The purchase of commercial sexual services should be prohibited.

These arguments highlight that there is something in the commercialization of sexual relationships and reproduction that is inherently harmful, and as such, that commercialization should be prohibited as a protective measure.

We can now compare the two sets of conclusions against one another to reveal the ethical inconsistency of New Zealand’s disparate approaches to regulating prostitution and commercial surrogacy. While one set of arguments concludes that individuals have the right to freely choose to work in the commercial sex industry or as gestational surrogates, the second set states that both the purchase of commercial sexual services and commercial surrogacy should be prohibited. These two conclusions are clearly inconsistent.

### Other Important Considerations

This essay has focused on the core ethical arguments foundational to New Zealand’s approaches to regulating the female body in prostitution and in commercial surrogacy. I have argued that they are ethically inconsistent approaches to regulating fundamentally similar practices of embodied labour. However, there are a number of other important considerations that may justify these disparate approaches. I explore two of these below.

#### The Importance of Whakapapa and Child Welfare

It is critical in discussions about the HART Act and its implications not to overlook another key principle of the Act: that “the needs, values, and beliefs of Māori should be considered and treated with respect” (s 4(f)). Assisted reproductive technologies have dramatically altered who can have children, how, and when, leading to a greater diversity of approaches to, attitudes towards, and laws surrounding family formation (Almond 2006). Lovelock (2010) explains that in most westernized countries and for Pākehā in New Zealand, alternate methods of family forming such as adoption have historically been conducted in an “as if” model. This seeks to integrate and socialize non-biological children “as if” they were biologically related. This was often accompanied by a policy of secrecy aimed at preventing the child from knowing who their “real” (biological) parents were. Māori, on the other hand, typically adopted openly from among biologically connected kin in whāngai arrangements in order to preserve the child’s whakapapa, a central aspect of personhood and identity in te ao Māori. In their work on Māori attitudes to assisted reproductive technologies, Glover and Rousseau (2007) found that many Māori saw reproduction of a child as reproduction of Māori as a whole, simply and beautifully summed up as “your child is your whakapapa.”

The HART Act section 63 contains requirements for information to be collected from all gamete and embryo donors and an additional provision that the donor’s whānau, hapū, and iwi to be recorded in the case of a Māori donor. The use of surrogates in New Zealand is relatively low. Between 2005 and 2010, only 104 applications for surrogacy were made. Despite provisions that show specific concern for the importance of whakapapa, the uptake of surrogacy among Māori has been lower than predicted, with Māori women comprising only 8.6 per cent of those willing to act as surrogates and 1.9 per cent of intended mothers (Anderson, Snelling, and Tomlins-Jahnke 2012). More research is required to understand the hesitancy toward surrogacy arrangements among Māori, including how the concept of whakapapa as it relates to a child’s holistic well-being is challenged or adapted in the context of gestational surrogacy.

Introducing a broader view of who is impacted in surrogacy arrangements beyond the gestational surrogate and intended parents—namely, the child—can help illuminate some of the key moral differences between commercial surrogacy and prostitution. Proponents of New Zealand’s current regulatory approach could plausibly argue that the procreative element of surrogacy leading to the creation of a new life is the morally relevant difference between the two practices. Under this view, allowing commercial surrogacy would be uncomfortably close to, if not indistinguishable from, purchasing a human life. The psychosocial impacts on the child’s well-being if or when their origins were discovered could be harmful. There are no analogous third parties whose welfare is so directly impacted through prostitution. That is, of course, only if it is carried out according to the PRA’s focus on safe sex practices that have the twofold intention of preventing sexually transmitted diseases and preventing pregnancies as the result of commercially purchased sexual services.

#### Using Different Methods to Achieve Similar Ends?

Perhaps the difference in regulatory approach stems from the existing state of the practices of sex work and surrogacy. In 2004, drafters of the HART Act were tasked with regulating new reproductive technologies before their emergence into mainstream use. Legislators, then, had the benefit of prohibiting a practice deemed unacceptable, commercial surrogacy, based upon a priori principles that the health and well-being of both children born as a result of commercial surrogacy and the women performing the gestating service would be harmed by the commercialization of procreation. In addition, based on an analysis of public submissions made on the HART Act, the range of ethical views on commercial surrogacy held by New Zealanders, particularly Māori, had yet to be deeply interrogated. Due to these significant unknowns, out of an abundance of caution in order to best fulfill the primary purpose of the HART Act, regulators may have chosen to utilize this opportunity to implement more restrictive policies that could be loosened in the future if it were to be shown that the country’s ethical views on commercial surrogacy had altered.

Prostitution, on the other hand, existed long before the PRA came into effect in 2003. Legislators were faced with implementing a dramatic overhaul of both existing legal precedent and longstanding moral and ethical views of the, although illegal, pre-existing and widespread practice of prostitution. Thus, while the two approaches look very different, it could be argued that in both cases the intention was to reduce the harms of each practice. This then leads to one further final question. Should we as citizens be concerned with the consistency of ethical principles underlying the legislation dictating the legality of our actions? Or, should we rather look to how effective a certain piece of legislation is in achieving its purposes? Should we prize principles or outcomes?

## Conclusion

In this essay, I have sought to elucidate the key ethical foundations underlying the approach taken to regulating prostitution and commercial surrogacy in New Zealand. The key difference in these views, I argued, stems from a disagreement in how to define the practices of sex work versus surrogacy. In New Zealand, sex work is understood as one of many forms of work, where workers are vulnerable to specific types of harm and exploitation. The PRA sought to provide sex workers with important health and safety protections and provide them with equitable access to justice for any infringements on their rights. This treatment of prostitution stands in stark contrast with how the practice is viewed by neo-abolitionists, who see it as an inherently harmful practice perpetuated by gendered power imbalances of our patriarchal society.

If commercial surrogacy were to be treated in the same manner as sex work in New Zealand, we would expect the practice to be decriminalized so that individuals could choose whether or not to engage in that form of work while being protected from exploitation. However, the prohibition of commercial surrogacy arrangements under the HART Act does not allow individuals to make this choice. There is an assumption that both the women acting as surrogates and the children born from a commercial surrogacy would be in some way harmed or disadvantaged in a way that those born from sexual intercourse, or even altruistic surrogacy, would not. While there is some evidence that children born as a result of surrogate pregnancies have increased adverse perinatal outcomes including, lower birth weight (Woo et al. 2017), there is nothing to suggest that the commercial exchange of funds in surrogacy would play a factor.

The most relevant potential harm to be protected against, then, must be the impact on the dignity of a child born from a commercial surrogacy arrangement. This would be a challenging impact to study but deserves attention if the claim of adverse impact is a key justification from limiting women’s free choice to engage in commercial surrogacy arrangements. An argument from the sex workers’ rights movement could be useful here: that it is the stigmatization of the practice that causes it to be harmful, rather than anything inherent in the practice itself. The legal prohibition of commercial surrogacy may in itself be a source of the perceived harm it seeks to prevent.

On the other hand, there may be something truly harmful about commercializing any part of the human experience once viewed as inalienable, whether that be sexual interaction or reproduction. In addition, as explored above, there is evidence that prostitution carries other significant negative impacts on those in the sex industry, including physical and sexual violence. Neo-abolitionists also stress the blurry line between those who truly choose to engage in sex work and those who are forced into the industry by coercion or the simple need to survive. A similar argument could be made for commercial surrogacy arrangements. A potentially lucrative form of employment, it may be likely to attract vulnerable or low-income women, who take on the medical risks of pregnancy and birth (Deonandan 2015).

In sum, I have argued that New Zealand’s approach to regulating the commodification of the female body in prostitution and commercial surrogacy are inconsistent in their ethical foundations. There are significant challenges facing those seeking to implement policy solutions based on either of the ethical foundations illustrated above. One key issue of regulating both prostitution and commercial surrogacy in a pluralistic, liberal democracy like New Zealand lies in ascertaining how the general public views each practice. Based on this analysis, I advocate for broader academic discussion and community consultation to interrogate whether there are additional morally significant reasons justifying what I see as an inconsistent legislative solution to two fundamentally similar issues.

## Footnotes

1 While the HART Act refers to “women,” people of many gender identities can become pregnant and may choose to act as gestational surrogates or utilize other assisted reproductive technologies. Similarly, people of all genders engage in sex work.

2 It should be noted that both of these estimates are likely outdated, in both cases based on data now over a decade old. In addition, both estimates noted significant barriers to obtaining accurate counts of sex workers as a marginalized population, even in a decriminalized context.

3 Subparagraph (a) states that trafficking of those over eighteen entails “the threat or use of force or other forms of coercion, or abduction, of fraud, of deception, of the abuse of power … for the purpose of exploitation.”

## References

[CR1] Abel G, Fitzgerald L (2008). On a fast-track into adulthood: An exploration of transitions into adulthood for street-based sex workers in New Zealand. Journal of Youth Studies.

[CR2] Abel G, Fitzgerald L, Brunton C (2009). The impact of decriminalisation on the number of sex workers in New Zealand. Journal of Social Policy.

[CR3] Almond B (2006). *The fragmenting family*.

[CR4] Anderson L, Snelling J, Tomlins-Jahnke H (2012). The practice of surrogacy in New Zealand. Australian and New Zealand Journal of Obstetrics and Gynaecology.

[CR5] Armstrong L, Fraser C, Armstrong L, Abel G (2020). The disclosure dilemma: Stigma and talking about sex work in the decriminalised context. *Sex work and the New Zealand model: Decriminalisation and social change*.

[CR6] Barnes J, Sutcliffe AG, Kristoffersen I (2004). The influence of assisted reproduction on family functioning and children's socio-emotional development: Results from a European study. Human Reproduction.

[CR7] Barnett T, Healy C, Reed A, Bennachie C, Abel G, Fitzgerald L, Healy C (2010). Lobbying for decriminalisation. *Taking the crime out of sex work: New Zealand sex workers’ fight for decriminalisation*.

[CR8] Belu D (2017). *Heidegger, reproductive technology, & the motherless age*.

[CR9] Benoit C, Smith M, Jansson M, Healhy P, Magnuson D (2019). “The prostitution problem”: claims, evidence, and policy outcomes. Archives of Sexual Behavior.

[CR10] Coy M, Smiley C, Tyler M (2019). Challenging the “prostitution problem”: Dissenting voices, sex buyers, and the myth of neutrality in prostitution research. Archives of Sexual Behavior.

[CR11] Deonandan R (2015). Recent trends in reproductive tourism and international surrogacy: Ethical considerations and challenges for policy. Risk Management and Healthcare Policy.

[CR12] Easterbrook-Smith G, Armstrong L, Abel G (2020). “Genuinely keen to work”: Sex work, emotional labour, and the news media. *Sex work and the New Zealand model: Decriminalisation and social change*.

[CR13] Farley M, Cotton A, Lynne J (2004). Prostitution and trafficking in nine countries: An update on violence and posttraumatic stress disorder. Journal of Trauma Practice.

[CR14] Glover M, Rousseau B (2007). “Your child is your whakapapa”: Māori considerations of assisted human reproduction and relatedness. Sites.

[CR15] Healy C, Pickering A, Hati C, Armstrong L, Abel G (2020). Stepping forward into the light of decriminalisation. *Sex work and the New Zealand model: Decriminalisation and social change*.

[CR16] Jordan J (2005). *The sex industry in New Zealand: A literature review*.

[CR17] Jordan J, Armstrong L, Abel G (2020). “On the clients’ terms”: Sex work before decriminalisation. *Sex work and the New Zealand model: Decriminalisation and social change*.

[CR18] Kesler K (2002). Is a feminist stance in support of prostitution possible? An exploration of current trends. Sexualities.

[CR19] Lewis S, Hofmann S, Moreno A (2016). Gestational labors: Care politics and surrogates’ struggle. *Intimate economies: Bodies, emotions, and sexualities on the global market*.

[CR20] Lovelock K (2010). Conceiving reproduction: new reproductive technologies and the redefinition of the kinship narrative in New Zealand society. Anthropological Forum.

[CR21] Mackenzie C, Scully JL, Baldwin-Ragaven LE, Fitzpatrick P (2010). Conceptions of autonomy and conceptions of the body in bioethics. *Feminist bioethics: At the center, on the margin*.

[CR22] Moran R, Farley M (2019). Consent, coercion, and culpability: Is prostitution stigmatized work or an exploitive and violent practice rooted in sex, race, and class inequity?. Archives of Sexual Behavior.

[CR23] New Zealand Prostitutes Collective. 2013. *Decriminalisation of sex work in New Zealand: Impact on Māori*. https://www.nzpc.org.nz/pdfs/Impact-on-Maori-Decriminalisation-of-Sex-Work-in-New-Zealand.pdf. Accessed June 2, 2022.

[CR24] Overall C (1992). What’s wrong with prostitution? Evaluating sex work. Signs.

[CR25] Park J, McLauchlan L, Frengley E (2008). *Normal humanness, change and power in human assisted reproductive technology: An analysis of the written public submissions to the New Zealand Parliamentary Health Committee in 2003*.

[CR26] Patrone T (2018). Is paid surrogacy a form of reproductive prostitution?. Cambridge Quarterly of Healthcare Ethics.

[CR27] Prostitution Law Review Committee (2005). *The nature and extent of the sex industry in New Zealand: An estimation*.

[CR28] Prostitution Law Review Committee (2008). *Report on the operations of the Prostitution Reform Act 2003*.

[CR29] Radin MJ (1987). Market inalienability. Harvard Law Review.

[CR30] Roberts D (2009). Race, gender, and genetic technologies: A new reproductive dystopia?. Signs.

[CR31] Sanders T (2016). Inevitably violent? Dynamics of space, governance, and stigma in understanding violence against sex workers. Studies in Law, Politics, and Society.

[CR32] Senent Julián R (2019). Tensions between feminist principles and the demand for prostitution in the neoliberal age: A critical analysis of sex buyer’s discourse. Recerca: Revista de Pensament i Anàlisi.

[CR33] Shamir H (2019). Feminist approaches to the regulation of sex work: Patterns in transnational governance feminist law making. Cornell International Law Journal.

[CR34] Showden C, Armstrong L, Abel G (2020). The future of feminism and sex work activism in New Zealand. *Sex work and the New Zealand model: Decriminalisation and social change*.

[CR35] Sullivan B, Caine B, Pringle R (1995). Rethinking prostitution. *Transitions: New Australian feminisms*.

[CR36] Tremblay F (2021). Labouring in the sex industry: A conversation with sex workers on consent and exploitation. Social Sciences.

[CR37] Turner J, Coy M (2012). Means of delivery: The trafficking of women into prostitution, harms and human rights discourse. *Prostitution, harm and gender inequality: Theory, research and policy*.

[CR38] United Nations General Assembly. 1948. *Universal declaration of human rights.* Paris. https://www.un.org/en/about-us/universal-declaration-of-human-rights. Accessed June 8, 2022.

[CR39] ____. 2000. *Protocol to prevent, suppress and punish trafficking in persons especially women and children, supplementing the United Nations convention against transnational organized crime.* https://www.ohchr.org/en/instruments-mechanisms/instruments/protocol-prevent-suppress-and-punish-trafficking-persons. Accessed June 8, 2022.

[CR40] *U.S. Department of State.* 2021. 2021 trafficking in persons report: New Zealand. https://www.state.gov/reports/2021-trafficking-in-persons-report/new-zealand/. Accessed June 8, 2022.

[CR41] Waltman M (2011). Sweden’s prohibition of purchase of sex: The law’s reasons, impact, and potential. Women’s Studies International Forum.

[CR42] Woo, I., R. Hindoyan, M. Landay et al. 2017. Perinatal outcomes after natural conception versus in vitro fertilization (IVF) in gestational surrogates: a model to evaluate IVF treatment versus maternal effects. *Fertility and Sterility* 108(6): 993-998. 10.1016/j.fertnstert.2017.09.014 29202976

